# Why do bacteria regulate public goods by quorum sensing?—How the shapes of cost and benefit functions determine the form of optimal regulation

**DOI:** 10.3389/fmicb.2015.00767

**Published:** 2015-07-28

**Authors:** Silja Heilmann, Sandeep Krishna, Benjamin Kerr

**Affiliations:** ^1^Computational Biology, Xavier lab, Memorial Sloan Kettering Cancer CenterNew York, NY, USA; ^2^National Centre for Biological Sciences, Simons Centre for the Study of Living MachinesBangalore, India; ^3^Department of Biology, University of WashingtonSeattle, WA, USA

**Keywords:** quorum sensing, cooperation, cost and benefit functions, bacteria, public good production and regulation

## Abstract

Many bacteria secrete compounds which act as public goods. Such compounds are often under quorum sensing (QS) regulation, yet it is not understood exactly when bacteria may gain from having a public good under QS regulation. Here, we show that the optimal public good production rate per cell as a function of population size (the optimal production curve, OPC) depends crucially on the cost and benefit functions of the public good and that the OPC will fall into one of two categories: Either it is continuous or it jumps from zero discontinuously at a critical population size. If, e.g., the public good has accelerating returns and linear cost, then the OPC is discontinuous and the best strategy thus to ramp up production sharply at a precise population size. By using the example of public goods with accelerating and diminishing returns (and linear cost) we are able to determine how the two different categories of OPSs can best be matched by production regulated through a QS signal feeding back on its own production. We find that the optimal QS parameters are different for the two categories and specifically that public goods which provide accelerating returns, call for stronger positive signal feedback.

## 1. Introduction

Bacterial cells live complex lives, constantly adjusting to the fluctuating presence or absence of nutrients, toxins, competitors and other environmental factors. They do this by regulating the production of different molecules that can perform the functions required to give them a fitness advantage in the current environment. Some of these molecules, like membrane bound nutrient receptors, are “private goods” which provide a benefit only to the individual cell that produced them. Others are molecules that are secreted by the cells and perform their function outside. Once secreted these molecules can diffuse away and potentially benefit other cells making them “public goods.” Microbes which produce extracellular molecules that can be thought of as public goods are ubiquitous. Examples of such products are: extracellular enzymes (Pirhonen et al., 1993), exopolysaccharides (used in biofilms) (Weiner et al., 1995; Vu et al., 2009), surfactants (aiding motility) (Kearns, 2010; Xavier et al., 2011), antimicrobial (for fighting other microbes) (Mazzola et al., 1992; Moons et al., 2005, 2006; An et al., 2006), virulence factors (for fighting a host organism's immune system, or for exploitation of host resources) (Zhu et al., 2002; Sandoz et al., 2007; Köhler et al., 2009), siderophores (for scavenging iron from the environment) (Neilands, 1984; Harrison and Buckling, 2009; Kümmerli and Brown, 2010). Although such cooperation in bacteria has received a lot of attention (Hense and Schuster, 2015), exactly how benefit and cost depends on the extracellular concentration of public good and the production rate of common goods (hereafter referred to as the benefit/cost function of the public good) has in general not been studied in great detail experimentally. Results from a recent theoretical paper (Cornforth et al., 2012), however shows that non-linearity in the benefit and cost curves is expected to radically impact the evolutionary dynamics of a cooperating population and that the shape of the benefit function will influence whether a population may benefit from having a public good under quorum sensing^1^ (QS) regulation. Bacterial public goods are in fact often under QS regulation. For example in *P. aeruginosa* secreted compounds are significantly overrepresented in the list of quorum sensing regulated gene products (Schuster and Greenberg, 2006). It is often assumed that public goods provide more benefit at higher population densities (e.g., Waters and Bassler, 2005; Diggle et al., 2007) and this has also recently been shown to be true experimentally for a specific public good produced by *P. aeruginosa* (Darch et al., 2012). There is however still a lack of general analytical arguments concerning exactly when and why a bacterial population may benefit from having a public good under QS regulation.

Here we use simple mathematical models, to find out how the shape of the cost and benefit function of a public good determines the optimal way to regulate the production of the public good. To illustrate our general point we have determined how the optimal public good production rate depends on the population size, for public goods with either accelerating or diminishing returns when cost is linear. Further we have determined how well a QS system can generate production curves that mimic the analytically determined optimal curves, and we discuss whether there might be evolutionary trade-offs associated with the optimal QS parameters for regulating certain public goods.

## 2. Models and results

### 2.1. Optimal production of public goods in a well-mixed population

We consider a well-mixed^2^ population of isogenic cells that produce a public good, *E*. Let Δ*g* denote the change in growth rate of such a population due to the act of producing the public good. Δ*g*, which could be negative or positive, can be decomposed into the benefits accrued from having the public good present and the costs of producing it. We make the following assumptions about the cost and benefit functions:

1. The cost is an increasing function of the rate of production of the public good.
2. The cost is zero when no public good is being produced.
3. The benefit is an increasing function of the concentration of the public good.
4. The benefit does not increase indefinitely as the concentration of the public good increases; it saturates at some finite value.
5. The benefit is zero when there is no public good present.

For now, we will assume that the cost is a linear function^3^ of the rate of production, σ_*E*_, of the public good, and the benefit a sigmoidal function of the concentration, *E*, of public good:

(1) $$\begin{array}{ll} \Delta g=benefit-cost\\ = & \beta_{1}\frac{{\left(E∕K_{E}\right)}^{h}}{1+{\left(E∕K_{E}\right)}^{h}}+\beta_{2}\frac{\left(E∕K_{E}\right)}{1+\left(E∕K_{E}\right)}-\kappa \sigma_{E} \end{array}$$

**Figure 1:**
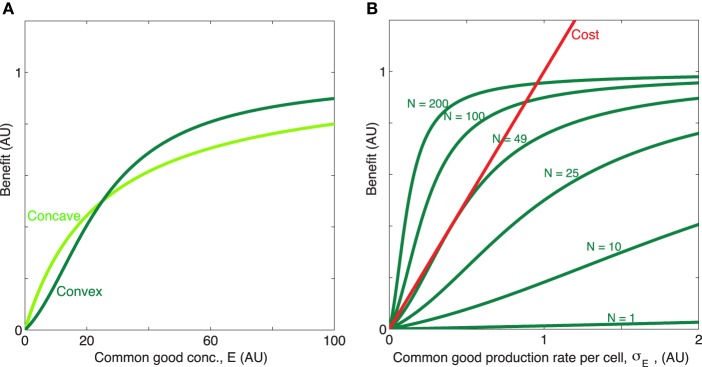
**Concave/convex benefit function**. **(A)** The benefit is here a sigmoidal function: $b\left(x\right)=\beta_{1}\frac{{\left(x∕K_{E}\right)}^{h}}{1+{\left(x∕K_{E}\right)}^{h}}+\beta_{2}\frac{\left(x∕K_{E}\right)}{1+\left(x∕K_{E}\right)}$, plotted for two different values of the exponent *h* = [1, 2], with *x* = *E*. The parameter *h* can be manipulated so that benefit initially decelerates (*h* = 1, concave, light green) or accelerates (*h* > 1, convex, dark green) with increasing concentration of public good. The remaining parameters are β_1_ = 0.7, β_2_ = 0.3, *K*_*E*_ = 25. **(B)** When the timescale of public good production and degradation are much faster than the timescale for growth of cells, public good concentration is proportional with production rate and population size, *E* ∝ σ_*E*_*N*. Dark green, the convex benefit curve *B*_*N*_(σ_*E*_) = *b*(*Nσ*_*E*_) is shown as a function of the common good production rate per cell σ_*E*_ for *N* = [1, 10, 25, 49, 100, 200]. Red, linear cost curve *c*(σ_*E*_) = κσ_*E*_, κ = 1.

**Figure 2:**
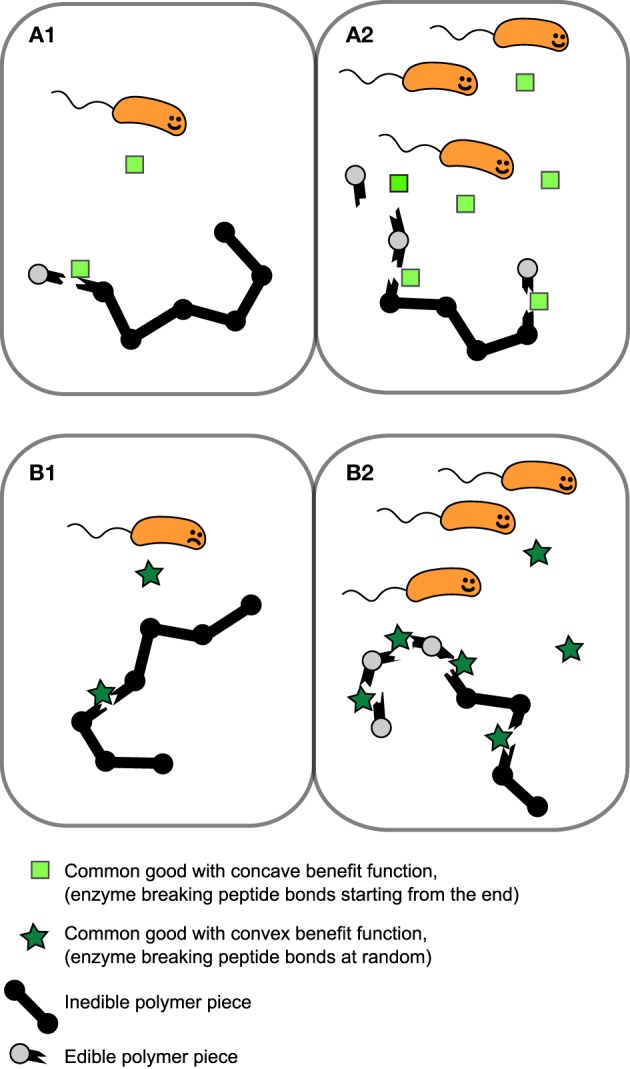
**Example of public goods with differently shaped benefit functions**. Proteases which works extracellularly by degrading polymers that are too large to be transported over the cell membrane can either break the peptide bonds of the polymer starting from the end of the polymer (exoprotease) or target a range of specific types of peptide bond within the chain, effectively breaking bonds at random (endoprotease). In this scenario benefit is proportional to the probability of yielding “edible” pieces of polymer. **(A1,A2)** Benefit increases approximately linearly with exoprotease concentration resulting in an initially concave benefit function. **(B1,B2)** The probability of breaking the polymer at a site producing an piece small enough to transport over the cell membrane is low when enzyme concentration is low, but accelerates as endoprotease concentration increases, resulting in a initially convex benefit function.

*K*_*E*_ is the concentration of of public good where the benefit is half of the maximum value, β_1_ + β_2_ is the maximum benefit possible and κ is the proportionality constant which quantifies how common good production translate to growth rate reduction. We will for now focus in particular on the shape of the first two terms of Equation (1), i.e., the benefit function of the public good, (see Figures 1A,B). The exponent *h* allows us to modulate the shape of the benefit function. When *h* ≤ 1, the benefit function is always concave, i.e., its slope is highest at *E* = 0 and steadily decreases as *E* increases. In other words, increasing public goods results in diminishing returns. In contrast, when *h* > 1, increasing public goods initially results in accelerating returns; i.e., the benefit function is initially convex. In Figure 2 we propose a concrete example of a class of public goods that can have differently shaped benefit functions depending on the precise molecular mechanism by which the public good works. Proteases that work by degrading proteins in the environment into smaller metabolizable pieces can work in two different ways: Exoproteases break the polymer peptide bonds starting from the end of the polymer and endoproteases target specific peptide bonds effectively breaking the polymers at random. These two modes of action can cause the benefit function to be either initially convex (endo) or concave (exo) (providing accelerating or diminishing returns, respectively. See supplement for the full length derivation which leads to this conclusion).

**Figure 3:**
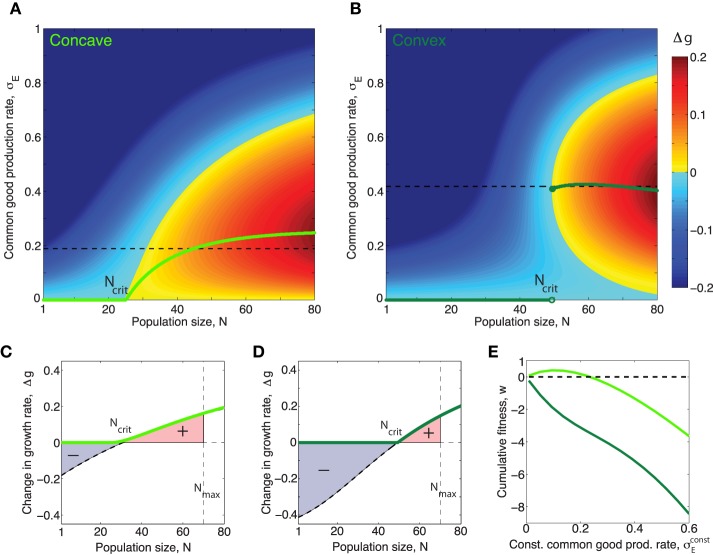
**Concave/convex benefit curves result in continuous/discontinuous optimal production curves respectively**. **(A,B)** Green curves show optimal production rate of public good, $\sigma_{E}^{opt}$ as a function of population size, *N* for the concave/convex benefit functions shown in figure 1. The optimal value, $\sigma_{E}^{opt}\left(N\right)$, corresponds to the σ_*E*_ which maximizes Δ*g* for *N*. The magnitude of Δ*g* in the (*N*, σ_*E*_)-space is shown by the colorbar. **(A)** In the case *h* = 1 the optimal production function is continuous and can be put in closed form: $\sigma_{E,h=1}^{opt}\left(N\right)=$ $\max\left(\sqrt{\left(\beta K_{E}\gamma_{E}\right)∕\left(cN\right)}-\left(K_{E}\gamma_{E}\right)∕N,0\right)$, where β = β_1_ + β_2_. The critical population size above which public good production is nonzero, when *h* = 1 is: $N_{crit}=\frac{c}{\beta }\gamma_{E}K_{E}$. **(B)** When the benefit curve is convex, *h* = 2, the optimal production function is discontinuous. **(C,D)**: Light/dark green curves show the effect on growth rate Δ*g* for a population producing common good at exactly the optimal rate when the benefit curve is concave/convex respectively. Black dashed curves show the effect on growth rate Δ*g* for a population producing common good at a constant rate $\sigma_{E}^{const}=1∕\left(N_{max}-N_{crit}\right)\Sigma_{N_{crit}}^{N_{max}}\sigma_{E}^{opt}\left(N\right)$, equal to the average of the non-zero part of the optimal curve, (shown as black dashed lines in **(A,B)**. **(E)** The cumulative fitness, $w\equiv \left(\int_{1}^{N_{max}}\Delta g\left(N\right)dN\right)∕w_{max}$, where $w_{max}\equiv \int_{1}^{N_{max}}\Delta g^{opt}\left(N\right)dN$, (def. in Equation fitnessDef) of different constant production strategies for a common good with concave (light green) and convex (dark green) benefit functions respectively. Note that in the case of the “concave common good” there is a range of different constant production rates which allows the population to perform better than a nonproducing population *w* > 0, however for the “convex common good” any constant production strategy will leed to worse fitness than that of a nonproducing population, *w* < 0 for all $\sigma_{E}^{const}>0$. (In this figure σ_*E*_ is given in units of κ∕γ_*E*_, which was set to one).

We now wish to determine how the convexity/concavity of the benefit function influences the optimal production strategy for a public good when the cost function is linear. The rate of change of the concentration of a public good *E* in a well-mixed system with *N* cells wil be given by:

(2) $$\begin{array}{l} \frac{dE}{dt}=N\sigma_{E}-E\gamma_{E}, \end{array}$$

where σ_*E*_ is the public good production rate per cell and γ_*E*_ is the degradation rate of the public good. For the sake of simplicity we will for now make the assumption that the timescales of public good production and degradation are much faster than the timescale for growth of cells. This allows us to assume that the public good concentration is always at steady state value E^*^ for a given population size:

(3) $$\begin{array}{l} E^{*}=N\frac{\sigma_{E}}{\gamma_{E}}, \end{array}$$

and thus we can express E^*^ as a function of σ_*E*_ When this assumption holds we can, by replacing *E* in Equation (1) with E^*^ from Equation (3) write the effect of the public good on the growth rate Δ*g*(σ_*E*_, *N*) in terms of population size *N* and production rate per cell σ_*E*_ only. In this situation, it is evident that cells which produce public good at a rate, $\sigma_{E}^{opt}$ that maximizes Equation (1), for each value of *N*, will grow fastest. In Figure 3 we have plotted this optimal rate $\sigma_{E}^{opt}\left(N\right)$ as a function of *N*. We see that the function $\sigma_{E}^{opt}\left(N\right)$ has very different properties when the benefit function is always concave (e.g., *h* = 1), compared to when it is initially convex (*h* > 1), even though the curves in Figure 1 appear quite similar at first glance. When *h* = 1, $\sigma_{E}^{opt}\left(N\right)$ rises smoothly from zero at *N*_*crit*_ to a maximum and then decreases smoothly to zero as *N* → ∞. In contrast, when *h* > 1, i.e., with accelerating returns, $\sigma_{E}^{opt}\left(N\right)=0$ for low values of *N*, jumps discontinuously to a non-zero value at *N*_*crit*_. After the jump the production rate may rise further to a maximum before decreasing to zero, or may simply decrease to zero, as *N* → ∞, depending on the values of other parameters such as *h*, β_1_, β_2_, *c*. For both a concave and a convex benefit curve (when cost is linear) the optimal behavior can be only to turn on common good production when a critical population size, *N*_*crit*_ > 1 has been reached^4^. The take home message is that the difference between the concave and the convex benefit curve cases is that in the convex case the optimal production curve will be discontinuous at the critical population size *N*_*crit*_^5^.

### 2.2. Regulating public goods using a quorum sensing system

In the previous paragraph we saw that the convexity/concavity of the public good benefit function is important for determining the shape of the optimal public good production curve, i.e., the way the optimal production rate depends on population size. Although the curves are very different it is clear that for both *h* = 1 and *h* > 1 the optimal production rate varies with the population size, (see Figures 3A,B) and that in both cases it thus looks like cells would benefit from having production linked to a mechanism which senses the cell density; a property which QS systems possess. We now wish to determine just how closely a QS regulation mechanism realistically could come to generating production curves matching the optimal curves in Figures 3A,B.

#### 2.2.1. A simple well-mixed model of QS regulated public good production

We formulate a simple ODE model of a well mixed population producing signal and public good with a positive feedback of signal on its own production:

(4) $$\begin{array}{rcl} \frac{dS}{dt} & = & N\left(1+\sigma_{S}^{max}\frac{{\left(S∕K_{S}\right)}^{\alpha }}{1+{\left(S∕K_{S}\right)}^{\alpha }}\right)-S \end{array}$$

(5) $$\begin{array}{rcl} \frac{dE}{dt} & = & N\sigma_{E}^{max}\frac{{\left(S∕K_{S}\right)}^{\alpha }}{1+{\left(S∕K_{S}\right)}^{\alpha }}-\gamma_{E}E \end{array}$$

Here *S* is the concentration of quorum sensing signal molecule^6^, *E*, the concentration of public good and *N* is the number of cells. The equations are non-dimensionalized by measuring all rates in units of the basal rate of signal production (i.e., the rate of signal production when signal concentration is very low) and by measuring time in units of the mean lifetime of a signal molecule 1∕γ_*S*_. The rate of signal production when the population is fully induced is $\sigma_{S}^{max}+1$ and^7^ $\sigma_{E}^{max}$ is the maximum rate of public good production per cell. The mean lifetime of a public good molecule is 1∕γ_*E*_ and *K*_*S*_ is the signal concentration where both signal production and public good production is at half of the maximum rate. The exponent α captures the strength of the positive feedback of the signal on its own production; for example this could be the cooperativity of the DNA binding by the transcription factor which activates production of signal synthase. In this model, signal receptor molecules are not modeled explicitly for the sake of simplicity. The parameter, α, does thus strictly not need to be interpreted just as the cooperativity of the transcription factor DNA binding but could also be influenced by the effect of signal positively feeding back on production of receptor molecules, (as discussed in Haseltine and Arnold, 2008; Rai et al., 2012). Assuming that both signal and public good have the same half-saturation constant *K*_*S*_ and feedback exponent α is probably a crude assumption which we will nonetheless make for the sake of simplicity.

**Figure 4:**
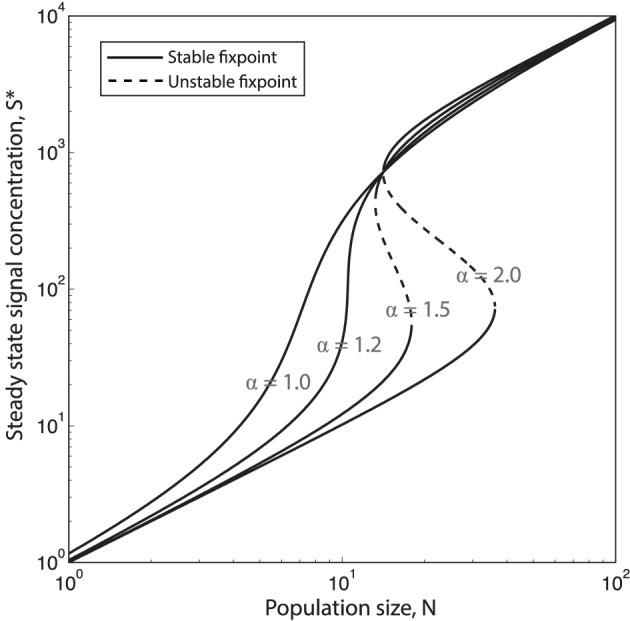
**Increasing the QS signal feedback exponent α causes hysteretic response to population changes**. Steady state QS signal concentration, S^*^ as function of the population size *N*, plotted for four different values of the QS feedback exponent α = [1.0, 1.2, 1.5, 2.0]. When $\alpha >\alpha_{c}=\frac{2+\sigma_{s}^{max}+2\sqrt{1+\sigma_{s}^{max}}}{\sigma_{s}^{max}}\approx 1.2210$ the system will be bistable for a certain range of population sizes. ($\sigma_{S}^{max}=100$, concentration of *S* is here given in units of $\sigma_{S}^{basal}∕\gamma_{S}$. $\sigma_{S}^{basal}$ is the basal production rate and γ_*S*_ is the degradation/depletion rate of the of signal, which is here set to 1.)

Once again we make the assumption that production and decay of quorum sensing molecules and public good molecules happen at a much faster timescale than growth of cells. This means that we can assume that for each population size *N*, signal concentration, *S*, will reach steady state, S^*^. In Figure 4 the steady state concentration of QS signal S^*^ is plotted as function of the population size *N* for different values of the feedback exponent α. We see that when α goes above a certain critical value α_*C*_ (see supplement for an analytical expression for α_*C*_) the system will have a bistable region with two stable equilibria, $S_{low}^{*}$ and $S_{high}^{*}$ (and one unstable, $S_{unstable}^{*}$ in between). Such bistability has indeed been observed in *Vibrio fischeri*: In Williams et al. (2008) they show that the regulation of expression of luxI during the *Vibrio fischeri* QS response exhibits hysteretic dependency on QS signal molecule (auto inducer) concentration [AI], due to AI-dependent autoregulation of luxR expression. They find that the “memory” of QS induction is maintained in the population due to a high level of LuxR, and thus a relatively high level of LuxR–AI complexes, even when [AI] is gradually decreased.

The public good production rate per cell, σ_*E*_, for a given population size *N* will be given by the first right hand side term in Equation (E) and thus depend on the steady state concentration of signal, S^*^(*N*):

(6) $$\begin{array}{l} \sigma_{E}\left(N\right)\equiv \sigma_{E}^{max}\frac{{\left(S^{*}∕K_{S}\right)}^{\alpha }}{1+{\left(S^{*}∕K_{S}\right)}^{\alpha }}. \end{array}$$

**Figure 5:**
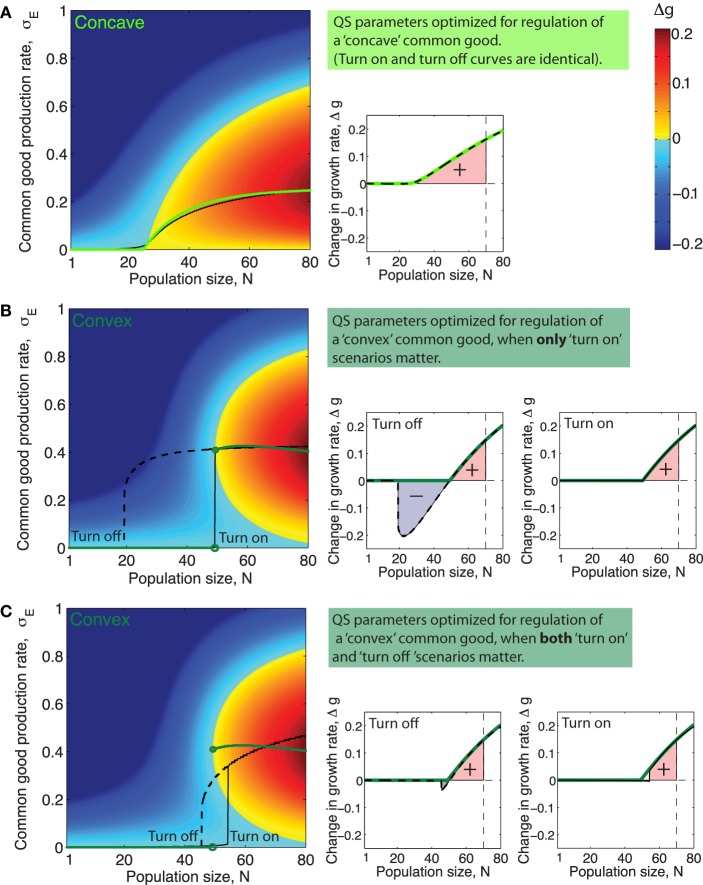
**Quorum sensing parameters optimized for different ecological scenarios and different types of public goods**. **(A–C)** Left side panels: Green curves shows optimal production rate, $\sigma_{E}^{opt}$, as a function of population size *N*. Black full line and dashed line show the production rate as a function of population size, *N*, when production is regulated by **(A)**: a QS system with parameters such that cumulative fitness, *w* is optimized for a public good with the concave benefit function shown in Figure 1 (α = 1.1, *K*_*S*_ = 2200, $\sigma_{E}^{max}=0.33$), **(B)**: a system with QS parameters for a public good with a convex benefit function maximizing cumulative fitness *w* in only “turn on” scenarios, (α = 2, *K*_*S*_ = 980, and $\sigma_{E}^{max}=0.43$), and **(C)**: a QS system with parameters that maximize cumulative fitness *w* for a public good with a concave benefit function in both “turn on” and “turn off” scenarios, (α = 1.4, *K*_*S*_ = 2600, and $\sigma_{E}^{max}=0.6$). **(A–C)** Right side panels: Green lines show Δ*g*^*opt*^, the increase in growth rate achieved, as a function of *N* when producing public good using the optimal production curve $\sigma_{E}^{opt}$. Full black line shows Δ*g* as a function of *N* for the QS regulated production curve marked “Turn on” in the plot to the right. Dashed black line shows Δ*g* as a function of *N* for the QS regulated production curve marked “Turn off” in the plot to the left. The colored area underneath the Δ*g* curves in the range *N* = [1, *N*_*max*_], (*N*_*max*_ = 70) is proportional to the cumulative fitness, *w* (defined in Equations 7 and 8).

When the system is in the bistable regime this means that there will be two different production curves; one which involves the lower stable equilibria ($S_{low}^{*}$) that the system will follow when going from low to high cell numbers and another curve which involves the the higher stable equilibria ($S_{high}^{*}$) which the system will follow when going from high cell numbers to low. Figures 5A–C (left panels) show examples of such QS production curves (with and without bistability) plotted together with the corresponding optimal curves.

### 2.3. Quantifying the fitness of a public good regulation strategy

Different choices of QS parameters α, *K*_*S*_ and $\sigma_{E}^{max}$ result in different public good production curves, σ_*E*_(*N*) and these curves, may resemble the optimal curves from Figures 3A,B more or less. In order to determine which QS parameters optimize the regulation of a public good with a specific benefit function we need a way to assess the fitness of a population of cells which utilize a specific “production curve” or in other words a specific “common good regulation strategy.” Equation (1) expresses how the impact of production on growth rate, Δ*g*, depend on the production rate, σ_*E*_. For a given QS regulated production curve σ_*E*_(*N*), Equation (1) gives a corresponding function Δ*g*(*N, h*) ≤ Δ*g*^*opt*^(*N, h*) which shows how growth is affected by public good production as population size, *N*, varies. In the right panels of Figures 5A–C, the corresponding Δ*g*(*N, h*) curve of the production curve σ_*E*_(*N*) from the left panel is plotted.

The cumulative fitness/performance of a given production curve/production strategy, σ_*E*_(*N*), can be quantified by the following expression:

(7) $$\begin{array}{l} w\equiv \frac{\int_{1}^{N_{max}}\Delta g\left(N\right)dN}{\int_{1}^{N_{max}}\Delta g^{opt}\left(N\right)dN}. \end{array}$$

This fitness measure quantifies how growth is affected by public good production over a range of different population sizes (the range set by the limits^8^ of the integral, [1, *N*_*max*_]). The cumulative fitness, *w*, is normalized with respect to the performance of a population which follows the optimal production strategy. This means that fitness will be one (*w* = 1) if the QS production curve perfectly mimics the optimal curve $\sigma_{E}^{opt}\left(N,h\right)$ and fitness will be zero (*w* = 0) for a population which does not produce public good at all, (negative fitness corresponds to having a lower fitness than a non-producing population).

#### 2.3.1. “Turn on” and “turn off” of public good production in the wild

It has been observed in several experimental studies (e.g., Sandoz et al., 2007) that, often non-producing cheater mutants will arise over time in populations which secrete a public good. Invasion of a faster growing cheater mutant could fragment the producer population and thus lead to an effective dilution of the wild type population. It seems that in this type of situation, having a mechanism which would down regulate production of public good in response to the decreasing producer cell density would be crucial for the wild type cells' ability to avoid population collapse (this scenario is also discussed in Melke et al., 2010). It thus seems there could exists ecological settings where both “turn on” and “turn off” scenarios are important for the fitness of the QS strategy.

Very little is known about actual ecological situations in the wild where bacteria use quorum sensing to regulate gene expression. In most of the quorum sensing literature the emphasis has been on the process of turning on QS regulated genes when population size increases, not off when it decreases. This bias might stem from the fact that “turn off” scenarios are probably not of great importance in the organism where QS was first discovered, *Vibrio fischeri*. This bacterium lives in symbiosis with the bobtailed squid (McFall-Ngai and Ruby, 2000; Visick et al., 2000; McFall-Ngai et al., 2012), an ecological setting where the bacteria periodically go through stages of population increase and decrease. The bacteria slowly grow to high density inside the light organ of the squid, reach the point where they turn on light production at nightfall only to be quickly diluted and turn off light production when the squid spurts out the majority of the bacteria by morning. In this situation, the dilution of signal and decrease in the population density happen so fast when the squid vents its light organ, that there is probably no need to have an accurate mechanism for down regulating light production as the population density decreases since very little time is spent at intermediate densities.

#### 2.3.2. Quantifying fitness in both “turn on” and “turn off” scenarios

As we saw earlier, there exist parameters for which the QS signaling system is bistable and thus where a set of QS parameters, ($\alpha ,K_{S},\sigma_{E}^{max}$), correspond to not one production curve but to two: one “turn on” curve which the population will follow when going from low to high numbers and one “turn off” curve which it will follow when going from high to low numbers. For a bacteria population that encounters both situations where they need to regulate public good expression as the population is increasing and when it is decreasing and which has such a bistable set of parameters, we thus need to assess the performance in both situations and include both in the fitness measure. One way of doing this is simply by using a weighted average of the cumulative fitness, *w*, of the two individual production curves:

(8) $$\begin{array}{l} w\equiv \theta_{on}w_{on}+\theta_{off}w_{off} \end{array}$$

[where the weights add up to one (θ_*on*_ + θ_*off*_ = 1)]. Depending on how often a population encounters “turn on” and “turn off” situations and on the relative importance of these situations the weight would be distributed differently. Since we do not have any information about what exactly these weights are for any actual ecological setting we will here, for the sake of argument, just compare the two extreme cases of (θ_*on*_ = 1, θ_*off*_ = 0) where strictly “turn on” scenarios are important for fitness (possibly an ecological setting like the case of *Vibrio fischeri* living in the bobtailed squid where population dilution happens very fast) and the case where “turn on” scenarios and “turn off” scenarios are equally important for fitness (θ_*on*_ = 0.5, θ_*off*_ = 0.5), (possibly an ecological setting where the population relatively often faces the risk of slow dilution due to the appearance of a cheater mutant).

#### 2.3.3. Fitness as a function of the QS feedback exponent, α

For a public good with a certain benefit/cost function pair there will be a specific set of QS parameters, (α, *K*_*S*_, $\sigma_{E}^{max}$), which maximizes the fitness, *w* (def. in Equations 7 and 8). This is, roughly speaking, the set of parameters that within the range [1, *N*_*max*_] manage to best mimic the optimal production curve (e.g., Figure 5A), or in the case where there is bistability and both “turn on” and “turn off” curves are considered to be equally important, it is the set of parameters which allows both curves to best mimic the optimal curve at the same time, (e.g., Figure 5C).

Often a single QS system will regulate the expression of many different secreted products that can be very diverse in nature and potentially have differently shaped benefit and cost functions. Since the parameter *K*_*S*_ quantifies the binding strength and $\sigma_{E}^{max}$ the overall promoter strength of the promoter regulating a specific gene product, it seems likely that these two parameters in most cases can be fine tuned by evolution to suit the regulation needs of individual QS products. The parameter α, on the other hand, characterizes the way the QS signal molecule feeds back on its own production and will thus in most cases be a parameter which has to be the same for all the QS regulated products, with the two exceptions that there can be more than one QS system in the cell and that it is possible to have different promoter sequences for signal synthase expression and public good production. For example LasR can bind either cooperatively or noncooperatively depending on the promoter sequence, Schuster et al. (2004). In Figures 5A–C left panels, the black lines show the QS regulated production curves where the choice of parameters $\sigma_{E}^{max}$, *K*_*S*_ and α maximize cumulative fitness, *w*, in three different situations. Interestingly, we see in Figure 5 that the feedback exponent α which optimizes fitness for a population regulating the “concave good” (*h* = 1) and the “convex good” (*h* = 2) are quite different. For *h* = 1 the optimal fitness of a QS production regulation curve is generally found at lower values of α, where there is no bistability, (in Figure 5A the optimal α value is around α ≈ 1). For *h* = 2 on the other hand, when fitness is optimized for “turn on” only, fitness increases monotonically with increasing α, approaching the maximal value of one, (note however that beyond α = 2 there is virtually no extra advantage to be had from increasing α further, so in Figure 5B the QS curve plotted simply has α = 2). At α values higher than α_*c*_ ≈ 1.2 we are in the bistable regime where the production curves will be different when going from “low to high” and “high to low” cell numbers. When *h* = 2 and fitness is optimized for both “turn on” and “turn off” the optimal feedback exponent is at an intermediate value α ≈ 3∕2. The reason for this is that at low values of α the QS production curve do not ramp up production fast enough while at higher values of α, the system becomes highly bistable and “turn on” and “turn off” curves thus very different which means they cannot both resemble the optimal curve.

#### 2.3.4. Regulating expression of both convex and concave public goods with one QS system

The α-values that optimize fitness for public goods with convex and concave benefit functions (when cost is linear) are different. It is thus interesting to think about what a bacteria population which needs to regulate several different types of public good with the same QS system would do. In general it is the case that a broader range of α-values will result in QS production curves with positive fitness for a concave good than for a convex, just as we saw in Figure 3E that a range of different constant production strategies resulted in *w* > 0 for the concave good, while all constant production startegies gave *w* < 0 for the convex good. This suggest that the evolutionary pressure which will drive the system towards the optimal α value could be stronger in the case of a convex good than for a concave good.

In Figures 5A–C left panels it is clear that for a concave good and a convex good the topology of the Δ*g*-“landscape” is quite different. Recall that different sets of QS parameters, (α, *K*_*S*_ and $\sigma_{E}^{max}$), correspond to different production curves: From at Figure 5A it is apparent that for a concave good there are several ways to “draw” suboptimal production curves through the Δ*g* “landscape” without encountering regions where Δ*g* ≪ 0, i.e., many choices of QS parameters which would give a positive cumulative fitness *w*. For *h* > 1 on the other hand we see that only curves which keep production relatively low for low *N* and then ramp up production very rapidly later can avoid passing through regions where Δ*g* ≪ 0. This means that for a convex good the range of QS parameters α, *K*_*S*_ and $\sigma_{E}^{max}$ that result in positive fitness is relatively narrow.

## 3. Discussion

### 3.1. Generalization to all cases of nonlinear cost and benefit function pairs

For the sake of simplicity we have throughout this paper considered the specific case of a linear cost function and two slightly different sigmoidal benefit functions, one concave and one convex (see Figure 1A). There is however no reason to believe that actual public goods will have costs and benefits which fit these arbitrarily chosen functions exactly. Fortunately it turns out that more general cases of nonlinear cost and benefit function pairs can be mapped onto the results presented. When cost is linear, we saw that the convex and the concave sigmoidal benefit functions fall into two major categories: Either they have continuous or discontinuous optimal production curves. It turns out that general pairs of (monotonically increasing) cost and benefit functions all fall into either of these two classes. We will refer to common goods with cost-benefit functions that cause them to have continuous optimal production curves as belonging to the “continuous class” and common goods with cost and benefit functions which cause them to have discontinuous optimal production curves as belonging to the “discontinuous class.” If the benefit function *b*(*Nσ*_*E*_) and the cost function *c*(σ_*E*_) for a certain common good are known, it is possible to determine whether the common good belong to the continuous or the discontinuous class. If

(9) $$\begin{array}{l} b^{-1}\left(y\right)\frac{db}{d\sigma_{E}}{|}_{0}>c^{-1}\left(y\right)\frac{dc}{d\sigma_{E}}{|}_{0} \end{array}$$

for all 0 < *y*, then the optimal production curve $\sigma_{E}^{opt}\left(N\right)$ is a continuous function.

While if there exists a value 0 < y^*^ such that

(10) $$\begin{array}{l} b^{-1}\left(y^{*}\right)\frac{db}{d\sigma_{E}}{|}_{0}<c^{-1}\left(y^{*}\right)\frac{dc}{d\sigma_{E}}{|}_{0} \end{array}$$

then the optimal production curve $\sigma_{E}^{opt}\left(N\right)$ has a discontinuity, causing it to jump from zero to a non zero value at *N* = *N*_*crit*_. See Supplementary Materials for the derivations of these criteria. It must be emphasized that as we expect the shape and magnitude of the cost and benefit functions to depend sensitively on specific growth conditions, we thus predict that the optimal production curves, and particularly the critical population size where production needs to be ramped up, to vary in different environmental conditions too. This prediction fits well with the observation by Duan and Surette (2007) that Las and Rhl expression profiles can vary greatly for different growth conditions. Such environmentally sensitive responce could for example be achieved by making QS parameters like *K*_*S*_ and $\sigma_{E}^{max}$, dependent on environmental cues such as nutrient availability and stress factors recently dubbed “pull” factors in the paper on core principles of QS systems by Hense and Schuster (2015).

### 3.2. Discontinuous class public goods require density dependent regulation

Overall we can draw the conclusion that when *N*_*crit*_ > 1 the discontinuous class of public goods (which resemble the case of a convex benefit curve and linear cost) require density dependent regulation more so than the continuous class of public goods, based on the properties of the optimal production curves. To reiterate, these properties are for a discontinuous class good:

1. The optimal production curve has a discontinuous jump from low to high production rate at *N*_*crit*_.
2. Even when *N* > *N*_*crit*_ production at a rate lower than the optimal one can cause a negative impact on fitness. (see Figure 5).

The optimal production curve of a continuous class good on the other hand have quite different properties:

1. It is possible to impact fitness negatively when *N* > *N*_*crit*_ by producing a continuous class good, but only by producing at too high a rate compared to the optimum, never when producing at a rate which is less than the optimum.
2. A group can indeed benefit from letting production of a continuous class good depend of population size but deviating from the optimal curve does not necessarily come at a great fitness cost.

Because of the different properties of the optimal production curves for continuous and discontinuous class goods the QS parameters which would be needed to regulate them differ too. In the case of a discontinuous class good, fitness can be negatively impacted if the QS feedback exponent is not appropriate. Conversely for a continuous class good, fitness can be positive for a broad range of QS feedback exponents and one could thus speculate that constant constitutive expression could be a better choice for regulation of a continuous class good than a potentially costly and elaborate QS regulation mechanism.

Interestingly Schuster et al. (2004) find that LasR can bind either cooperatively or non-cooperatively depending on the promoter sequence. So although the overall signal feedback is characterized by a single exponent α_*S*_, the individual public goods, *E*_*i*_, can have different responses to the signal-receptor complex characterized by different exponents α_*E*_*i*__ (Schuster et al., 2004). The reason for these differences could perhaps be that the various secreted products have different cost/benefit functions and thus different optimal production curves and consequently different regulation needs.

### 3.3. QS signal feedback

Quorum sensing is a mechanism usually assumed to give individual cells information about the density of the population. It is thus paradoxical that one feature found in many quorum sensing systems, the positive signal feedback, actually makes a system less accurate for sensing population size changes. Roughly speaking a strong positive signal feedback makes a QS system more appropriate for answering the binary question “are we many or few?” than for providing information about the precise population size over a broad range of densities. The reason usually given for why QS signals often feed back positively on their own production, is that this feedback ensures a synchronized response across a population (Hense and Schuster, 2015). Our analysis inspires another explanation for the existence of positive QS signal feedback, which does not however exclude the existing one. Recall that for a discontinuous class public good the optimal production strategy calls for a sudden discontinuous jump in production rate at a critical population size *N*_*crit*_. After this critical population size has been reached a substantial growth increase can be gained by producing public good at a specific optimal rate, but there will exist a production rate lower than the optimal one which will result in a growth rate decrease (see Figures 5B,C, for *N* > *N*_*crit*_ there exists a local fitness minima below the optimal rate $\sigma_{E}^{min}<\sigma_{E}^{opt}$). When ramping up production from zero to the optimal rate, the population thus necessarily has to pass through a local fitness minimum. A reason for having a strong positive signal feedback could thus be to ensure a sharper turn on of the public good production in order to minimize the time spent at the low production rates where fitness is impacted negatively.

### 3.4. Potential trade offs

When only “turn on” scenarios are important for fitness it is just the production curve that the population follows when going from low to high numbers, which needs to mimic the optimal curve, and bistability does not matter. If however both “turn on” and “turn off” scenarios are important for fitness, this changes. Now both the production curve which the population follows going from low to high numbers and the curve it follows from high to low numbers needs to mimic the optimal one simultaneously. For higher values of the feedback exponent this becomes problematic since the two curves will differ more and more due to the increasing width of the bistable range. This suggests that a bacterial species which needs to accurately regulate the production of a discontinuous class good both in situations where the population increases and decreases, will face an evolutionary trade off between “precision” and “sharpness” of the production curves. Too high a feedback exponent will mean imprecise turn on/off points for either (or both curves) due to the bistability and too low a feedback will mean that the turn on/turn off of the public good does not happen sharply enough. We thus predict that a bacteria living in such an ecological settings will have a feedback exponent bound at intermediate values—relatively close to the point where the QS system starts to display bistability.

### 3.5. Ideas for experiments

With few exceptions (Melke et al., 2010; Sappington et al., 2011) there is a general tendency in the QS literature to focus only on the “turn on” of QS genes and only few studies have looked thoroughly at the effect of positive signal feedback (in fact it is disregarded in some theoretical studies, Brown and Johnstone, 2001; Nadell et al., 2008; Pai and You, 2009). To our knowledge no one has attempted to assess the fitness cost of bistability when both “turn on” and “turn off” scenarios are important. Our analysis suggest thats it could be interesting to explore experimental setups which examine how QS regulated genes are turned off in response to a decrease in population density, and setups which probe the effect of signal feedback on bistability. For example our results suggest that two strains engineered to differ in their QS feedback exponents would perform differently when placed in the experimental setup of a turbidostat where controlled slow fluctuations in density was set to occur right around the point of QS turn-on and turn-off. A separate treatment could involve major quick dilution and growth well past the turn-on point. In the first case, both turn-on and turn-off would be relevant, whereas in the second case, turn-on would be more important.

#### 3.5.1. Measuring the shape of public good cost and benefit functions

The benefit function of a public good (e.g., elastase produced by *P. aeruginosa* Pearson et al., 1997; Beaufort et al., 2013) could be quantified by measuring the growth rates of signal-blind cheats (e.g., the lasRrhlR mutant) in a chemostat, as a function of the concentration of externally added public good (e.g., LasB, which would act as a public good by facilitating the break down of the main source of carbon which would be in the form of casein peptide chains to large for transport across the cell membrane). The cost function could be quantified by measuring the growth rate of an inducible constitutive producer mutant at different expression rates. It would be interesting to automate these types of measurements so that they could be done for a wide range of different molecules thought to be public goods, from different bacterial species. The shape/convexity of the measured benefit and cost functions could then be compared with already known information about whether the molecules are QS regulated or not, to determine whether discontinuous class public goods are overrepresented among QS regulated compounds.

#### 3.5.2. Manipulating the shape of the benefit function experimentally

The way the specific public good LasB of *P. aeruginosa* works might provide a way to manipulate the convexity of its benefit function. When provided solely with a diet of casein polymers, *P. aeruginosa* growth depends on the production of LasB (and similar proteases) that degrade the casein polymers into smaller importable units, which can be transported across the cell membrane and metabolized (Efrat and Mary, 2014). In the Supplementary Materials we show that the benefit function becomes increasingly convex if the maximum length of the polymers in the environment is increased. This suggests that one way of experimentally manipulating the benefit function would be to pre-digest casein polymers to varying degrees before providing them to *P. aeruginosa*. Media with undigested casein should result in a more convex benefit function than media with pre-digested casein. Growth of constitutive producer mutants could then be compared with growth of wild-type *P. aeruginosa* in these media to test our predictions regarding the importance of the convexity of the benefit function for QS regulation.

### Conflict of interest statement

The authors declare that the research was conducted in the absence of any commercial or financial relationships that could be construed as a potential conflict of interest.

## Footnotes

^1^Quorum sensing (QS) is a bacterial behavior ubiquitously present in the microbial world and most bacteria possess at least one quorum-sensing system, (Fuqua et al., 2001; Miller and Bassler, 2001; Schauder and Bassler, 2001). This term covers all types of behavior where bacteria produce, secrete, and subsequently respond to diffusable signal molecules. Typically, the signal molecules are small and relatively cheap to produce, e.g., an acyl homoserine lactone (Fuqua et al., 2001).

^2^Using a well-mixed system is a simplification that allows us to treat each cell as identical to all others and to ignore spatial variations in the public good concentration.

^3^More on general nonlinear cost and benefit functions in the discussion.

^4^If *N*_*crit*_ < 1 even a single cell will obviously benefit from production no matter what the benefit curve is shaped like

^5^See Supplementary Materials for a further discussion on (1) the case of a spatially structured system, (2) situations where the assumption that timescales of production and decay of public good are much faster than the time scale of growth does not hold.

^6^Actually the feedback acts not on the signal production but on the production of the signal synthase, an enzyme which facilitates the production of signal intracellularly, but since concentration of signal synthase and signal has been found to be approximately proportional (e.g., in the Las system of *P. aeruginosa* Duan and Surette, 2007), we can model concentration of signal and signal synthase by one symbol *S*.

^7^Note that because all rates are given in units of the basal signal production rate ($\sigma_{S}^{basal}\equiv 1$), $\sigma_{S}^{max}$ is in fact the ratio between the basal rate and the maximum rate.

^8^For this study we examine the behavior of the system in the vicinity of the critical turn on point, *N*_*crit*_ and not in the limit of high *N*. As a population approaches stationary state or starvation additional layers of gene regulation will become relevant and cause down regulation of genes which have been activated by QS, (Navarro Llorens et al., 2010; Gopalkrishnan et al., 2014). This type of regulation was not included in our simple QS model which is the reason that we choose to evaluate the QS production curves as a function of population size only on in the range [1; *N*_*max*_] where *N*_*max*_ is not much larger than the critical population size *N*_*crit*_ above which cooperation become beneficial.
