# Supplementary material for: Why do bacteria regulate public goods by quorum sensing?—How the shapes of cost and benefit functions determine the form of optimal regulation
Source: Front Microbiol. 2015 Jul 28;6:767. doi: 10.3389/fmicb.2015.00767 (PMC4517451; doi:10.3389/fmicb.2015.00767)
Supplement: Supplementary file 1 [file SupplementalMaterials.PDF]

Supplementary material for: "Why do bacteria regulate public goods by quorum sensing? - How the shapes of cost and benefit functions determine the form of optimal regulation"

Silja Heilmann, Sandeep Krishna & Benjamin Kerr

June 11, 2015

1 Explanation of units and parameter choices for figures

For easy referral here are equation (1), (4) and (5) from the main text:

$$\begin{aligned}\Delta g &= \quad benefit \quad - \quad cost \\ &= \beta_1 \frac{(E/K_E)^h}{1+(E/K_E)^h} + \beta_2 \frac{(E/K_E)}{1+(E/K_E)} - \kappa\sigma_E\end{aligned}$$

$$\begin{aligned}\frac{dS}{dt} &= N\left(1+\sigma_S^{max}\frac{(S/K_S)^\alpha}{1+(S/K_S)^\alpha}\right) - S \\ \frac{dE}{dt} &= N\sigma_E^{max}\frac{(S/K_S)^\alpha}{1+(S/K_S)^\alpha} - \gamma_E E\end{aligned}$$

See table 1 below for for a full list of all the variables and parameters, with units and explanations as well as the specific values or value ranges used in the figures.

1.1 Reduction of the number of free parameters

To reduce the number of free parameters we choose units of time and concentration in terms of parameters of the system, (aka. set two selected parameter values, γ_E and κ to one). Setting $\gamma_E \equiv 1$ is equivalent to setting the time unit to be equal to the characteristic lifetime of a common good molecule: $t' \to t = t'/\tau_E$ (where $\tau_E = 1/\gamma_E$). Setting $\kappa \equiv 1$ is equivalent to setting the unit of concentration to be equal to $1/\kappa$ such that $E' \to E = E'\kappa$. (Recall that κ is the proportionality constant (unit $[1/conc.]$) between cost (negative impact of growth rate, unit $[1/time]$) and common good production rate (unit $[conc./time]$). Setting $\gamma_E = 1$ and $\kappa = 1$ means that the common good production rate σ_E is measured in units of γ_E/κ, and a $\sigma_E = 1$ thus means that a cell is producing an amount of common good within the time span of $\tau_E = 1/\gamma_E$ such that its growth rate is reduced by exactly $\Delta g = \gamma_E$.

**Parameter/variable**	**Explanation**	**Unit**	**Value/range used**
γ_E	Degradation/depletion rate of common good molecule	$[1/time]$	Set to one (thus setting the unit of time)
κ	Proportionality constant between cost and common good production rate σ_E	$[1/conc.]$	Set to one (thus setting the unit of concentration)
Δg	Net change in growth rate of population as a results of common good production	$[1/time]$	$[-0.2, 0.2]$
Benefit, $b(N, \sigma_E)$	Positive impact on growth rate due to presence of common good	$[1/time]$	$[0, 1]$
Cost, $c(\sigma_E)$	Negative impact on growth rate due to production of common good	$[1/time]$	$[0, \infty[$
β_1, β_2	$\beta_1 + \beta_2$ is the maximum possible benefit. β_1 and β_2 - the weights of each of the sigmoidal terms in the benefit function	$[1/time]$	$\beta_1 = 0.7$ and $\beta_2 = 0.3$
N	Integer number of cells in system	unitless	$[1, 80]$
σ_E	Production rate of common good	$[conc./time]$	$[0, 1]$
K_E	Conc. of common good where benefit is half the maximum value	$[conc.]$	25
K_S	Conc. of QS signal molecule where both signal production and public good production is at half of the maximum rate	$[conc.]$	1
γ_S	Degradation/depletion rate of QS signal good molecule	$[1/time]$	1
$\sigma_S^{basal} + \sigma_S^{max} \frac{S^\alpha}{S^\alpha + K_S^\alpha}$	Production rate of QS signal molecule	$[conc./time]$	$[1, 100]$
σ_S^{basal}	Basal rate of QS molecule production when there is no signal molecules in the environment	$[conc./time]$	1
σ_S^{max}	$\sigma_S^{basal} + \sigma_S^{max}$ is the maximum rate of QS signal molecule production when bacteria are fully induced	$[conc./time]$	100
h	Allows us to modulate the shape of the benefit function from concave to convex	unitless	1 and 2
α	The strength of the positive feedback of the QS signal on its own production	unitless	$[1, 2]$
E	Conc. of common good molecule	$[conc.]$	$[1, 100]$
S	Conc. of QS signaling molecule	$[conc.]$	$[1, 10^4]$

Table 1: List of variables and parameters in eq. (1), (4) and (5)

1.2 Choice of parameter values used in plots

The choices for the values of the parameters $\beta_{1,2}$ and K_E were fairly arbitrary. We chose to set β_1 and β_2 such that $\beta_1 + \beta_2 = 1$ such that the benefit function saturates at one. (Note that unit of benefit is also $[1/time]$ since it measures increase in growth rate, so with $\gamma_E = 1$ this means that the maximal positive change in growth rate possible is equal to γ_E. This choice is completely arbitrary, as the saturation value of the benefit function is not believed to be connected to the degradation rate of the common good!). The less weight we put on the first term of the benefit function (the term with β_1), the more similar the two example functions (concave, $h = 1$ and convex, $h = 2$) used in the text become. We simply picked β_1 and β_2 such that the concave and convex curves were similar enough that the reader would recognize that even subtle changes in shape matter but not so similar that the curves would be overlapping and hard to distinguish between visually.

For our choice of cost function and max benefit value ($\beta_1 + \beta_2 = 1$) we wanted to pick a K_E, which would mean that the critical population size above which cooperation pays off was greater than $N = 1$ cell for both $h = 1$ and $h = 2$. For the arbitrarily chosen $K_E = 25$ this was the case: $N_{crit}(h = 1) \approx 25$ cells and $N_{crit}(h = 2) \approx 50$ cells.

We set the decay/depletion rate of the signal to one ($\gamma_S = 1$). Note that we are thus assuming that decay/depletion rate of common good and signal molecules are the same which is not necessarily the case - this is a choice we made for the sake of simplicity.

2 Assumptions about cost and benefit functions

We assume that growth of bacteria happens at a much slower time scale than production and decay of common good and we also assume that the population is well-mixed and isogenic. This means that the external common good concentration will approx. reach a new steady state each time the population increases by one. In this type of situation the steady state concentration of common good is proportional to the single cell production rate of common good and the population size, $E \propto \sigma_E N$, and the benefit, which is a function of the common good concentration, $b(E)$, can thus just as the cost function be written as a function functions of the common good production rate, σ_E and the population size, N:

$$\begin{aligned} B_N(\sigma_E) &= b(N\sigma_E) \\ C_N(\sigma_E) &= c(\sigma_E) \end{aligned}$$

We will assume that benefit, b and cost, c are continuous and continuously differentiable functions and that there is no cost or benefit associated with zero production of common good such that:

$$b(0) = c(0) = 0$$

Fitness impact Δg of common good production (e.g. in the form of increase/decrease of growth rate) is given by: $\Delta g = b - c$ and is thus a function of σ_E. For each N there will be an optimal production rate $\sigma_E^{opt} \geq 0$ which maximizes Δg. Firstly we wish to determine when the optimal production rate $\sigma_E^{opt}(N)$ is zero and when it is non-zero.

3 Determining the critical population N_{crit} size where common good production starts to pay off

It is beneficial or neutral for the population to produce common good at a nonzero rate σ_E whenever N is such that benefit is greater or equal to cost, $b(N\sigma_E) \geq c(\sigma_E)$. See figure 1 - the effect of increasing N is that the curve $B_N(\sigma_E) = b(N\sigma_E)$ is pushed upwards and leftwards as the function b is squeezed due to the effective rescaling of the x-axis. At a critical population size, N_0, the benefit and cost curves are rising from $\sigma_E = 0$ at the same rate:

$$\left.\frac{dB_{N_0}}{d\sigma_E}\right|_0 = \left.\frac{dC_{N_0}}{d\sigma_E}\right|_0 .$$

This happens at:

$$N_0 = \frac{\left.\frac{dc}{d\sigma_E}\right|_0}{\left.\frac{db}{d\sigma_E}\right|_0}.$$

Since if we let $u(\sigma_E) = N\sigma_E$ and use the chain rule we have:

$$\left.\frac{dB_N}{d\sigma_E}\right|_0 = \left.\frac{db(N\sigma_E)}{d\sigma_E}\right|_0 = \left\{\left.\frac{db(u)}{du}\right|_{u=0}\right\}\left\{\left.\frac{du}{d\sigma_E}\right|_{\sigma_E=0}\right\} = \left\{\left.\frac{dB_1}{d\sigma_E}\right|_0\right\}N = \left\{\left.\frac{db}{d\sigma_E}\right|_0\right\}N.$$

For population sizes $N > N_0$ common good production can be beneficial since here benefit must be greater than cost for production rates infinitesimally close to zero, so the optimal production rate must be nonzero, $\sigma_E^{opt} > 0$ for $N > N_0$.

But can there be an even smaller population size $N_{crit} < N_0$ above which common good production pays off? And if yes can this value be defined in terms of b and c?

Both b and c are invertible, so we may define the function:

$$R(y) = \frac{b^{-1}(y)}{c^{-1}(y)}$$

on the domain $y \in]0, \infty[$. Let the common good production rate be given by $\sigma_E^* > 0$. There must exists a population size N^* such that cost and benefit curves intersect at (σ_E^*, y^*):

$$B_{N^*}(\sigma_E^*) = c(\sigma_E^*) = y^* \tag{1}$$

or equivalently

$$B_{N^*}^{-1}(y^*) = c^{-1}(y^*) = \sigma_E^* \tag{2}$$

Note that since $B_N(\sigma_E) = b(N\sigma_E)$ the inverse functions B_N^{-1} and B_1^{-1} differ only by a factor N when evaluated in the same value y, e.g.:

$$B_{N^*}^{-1}(y^*) = \sigma_E^* \qquad \text{and} \qquad B_1^{-1}(y^*) = N^*\sigma_E^* \tag{3}$$

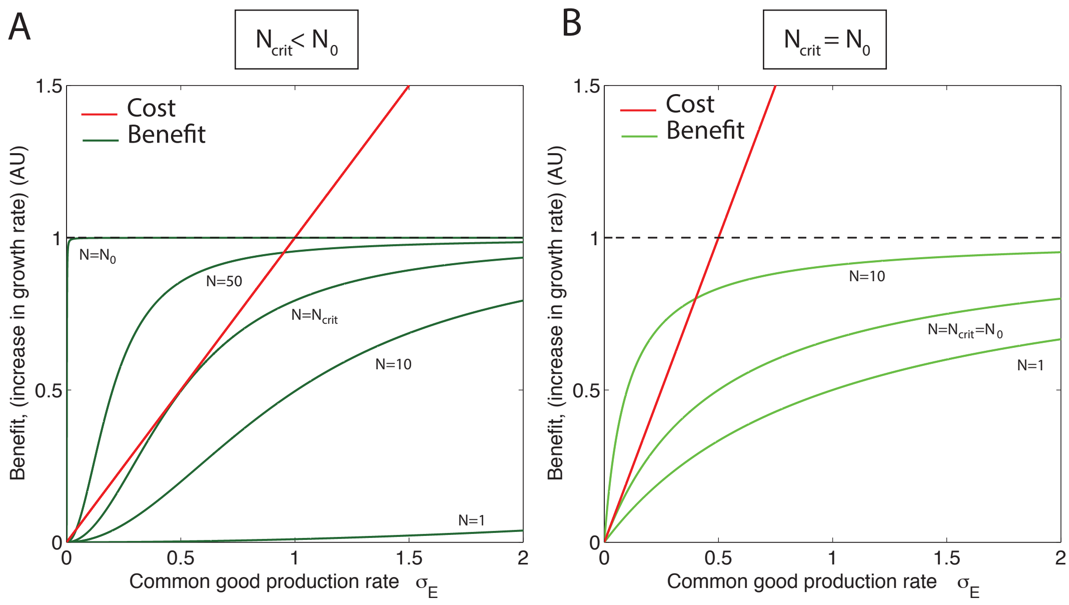

Figure 1: **A**: an example of a case 1. scenario, where $N_{crit} < N_0$. **B**: an example of a case 2. scenario where $N_{crit} = N_0$.
Red curves show $c(\sigma_E) = \text{const} \cdot \sigma_E$ and green curves show $B_N(\sigma_E) = b(N\sigma_E)$ for different values of N. Where $b(x) = b_1 \frac{(x/K_E)^h}{1+(x/K_E)^h} + b_2 \frac{(x/K_E)}{1+(x/K_E)}$, with $b_1 = 0.7$, $b_2 = 0.3$, $K_E = 25$ A: $h = 2$, B: $h = 1$.

Using eq. (2) and eq. (3) we can rewrite the newly defined function $R(y^*)$ as:

$$\begin{aligned} R(y^*) &= \frac{b^{-1}(y^*)}{c^{-1}(y^*)} \\ &= \frac{B_1^{-1}(y^*)}{B_{N^*}^{-1}(y^*)} \\ &= \frac{N^*\sigma_E^*}{\sigma_E^*} \\ &= N^* \end{aligned}$$

We see that $R(y^*)$ is equal to the population size N^* at which a group producing common good at the nonzero rate σ_E^* will feel exactly equal cost and benefit levels. The global minimum of $R(y)$ (if such exists) must thus be the smallest possible population size where benefit and cost can be equal for a nonzero production rate, and thus the critical population size above which the population will benefit from common good production.

There can thus be two types of cases:

1. If a value $y^* > 0$ exists such that $R(y^*) < N_0$, $R(y)$ has a global minimum and the critical population size above which production should start is at this minimum: $N_{crit} = \min(R(y))$.
2. If $R(y) > N_0$ for all $y > 0$ there is no global minimum of $R(y)$ and the critical population size above which production should start must simply be: $N_{crit} = N_0$.

We are only interested in knowing the form of the optimal production function $\sigma_E^{opt}(N)$ in the biologically relevant domain of population sizes of one cell and up, i.e. $N \in [1, \infty[$. If $N_{crit} < 1$ the situation is simple: the optimal production function is always nonzero even for a single cell no matter what the functions b and c look like. In cases where $N_{crit} > 1$ common good production does not pay of at the single cell level - the optimal production function $\sigma_E^{opt}(N)$ starts at zero and only becomes nonzero above N_{crit}. We now wish to determine whether $\sigma_E^{opt}(N)$ rises continuously or discontinuously from zero at N_{crit}, in case 1. ($N_{crit} < N_0$) and case 2. ($N_{crit} = N_0$).

4 Criteria for continuity / discontinuity of the optimal production function

4.1 Case 1.

Say we have common good which satisfies criteria of case 1. (i.e. a value $y^* > 0$ exists such that $R(y^*) < N_0$). Since $R(y^*) = N^* < N_0$ we can pick an value N^{**}, between N^* and N_0 (e.g. $N^{**} = \frac{N_0+N^*}{2}$) where for σ_E infinitesimally close to zero we have $c < B_{N^{**}}$, but where:

$$B_{N^{**}}(\sigma_E^*) = b(N^{**}\sigma_E^*) > b(N^*\sigma_E^*) = B_{N^*}(\sigma_E^*) = c(\sigma_E^*).$$

This means that $\sigma_E^{opt}(N)$ must be a **discontinuous function**. It starts at zero for $N = 1$ and must be strictly positive for some N^{**}; however it cannot take on any values

infinitesimally close to zero for any $1 \leq N \leq N^{**}$. A common good with a benefit cost function pair b, c that satisfies Case 1 is thus equivalent to what in the main text is referred to as the 'discontinuous class of common goods'.

4.2 Case 2.

If on the other hand, if we have a common good which satisfies the criteria for case 2., i.e. $R(y) > N_0$ for all $y > 0$ then we know that there exists $\epsilon > 0$ such that for $N_0 + \epsilon$, $\sigma_E^{opt} > 0$ where ϵ can be infinitesimally small.

Assume now that $\sigma_E^{opt}(N)$ is discontinuous. Then there must exist values δ and N^{***}, where $\delta > 0$ and $N^{***} \leq N_0 + \epsilon$, such that:

$$B_{N^{***}}(\delta) = c(\delta)$$

and N^{***} is the point of discontinuity in $\sigma_E^{opt}(N)$, specifically:

$$\begin{aligned}
\sigma_E^{opt}(N) &= 0 \text{ for } N < N^{***} \\
\sigma_E^{opt}(N) &> 0 \text{ for } N > N^{***} \\
\lim_{N \to N^{***}(+)} \left(\sigma_E^{opt}(N)\right) &= \theta > 0 \\
\lim_{N \to N^{***}(-)} \left(\sigma_E^{opt}(N)\right) &= 0
\end{aligned}$$

(Where $(+)$ and $(-)$ indicate limits from the right and left respectively). Let $y^{***} = c(\delta)$. If $N^{***} < N_0$ then this would imply that

$$B_1^{-1}(y^{***}) = b^{-1}(y^{***}) = N^{***}\delta \leq N_0\delta = N_0 c^{-1}(y^{***})$$

which implies

$$R(y^{***}) \leq N_0$$

which violates our assumption that $R(y) > N_0$ for all $y > 0$. So

$$N_0 < N^{***} \leq N_0 + \epsilon$$

However, for any $N^{***} = N_0 + \epsilon'$ (where $0 < \epsilon' \leq \epsilon$) in this range we can always pick a new value ϵ'', where $0 < \epsilon'' < \epsilon'$, such that for $N_0 + \epsilon''$, $\sigma_E^{opt} > 0$, which violate the condition that N^{***} is the point of discontinuity. This contradiction implies that we must have a **continuous function** for σ_E^{opt}. A common good with a benefit cost function pair b, c that satisfies Case 2 is thus equivalent to what in the main text is referred to as the 'continuous class of common goods'.

5 Examples of cost benefit function pairs falling into the continuous and discontinuous categories

Consider the following non linear cost benefit function pair:

$$c(\sigma_E) = \sigma_E^{h_c}$$

$$b(\sigma_E) = (\sigma_E)^{h_b}$$

Where $h_c, h_b > 0$ and $h_c \neq h_b$.

Recall that the criteria for having a continuous optimal production function is that for all $y > 0$:

$$b^{-1}(y) \left. \frac{db}{d\sigma_E} \right|_0 > c^{-1}(y) \left. \frac{dc}{d\sigma_E} \right|_0$$

(eq. (9) in the main text), which can be rewritten:

$$\frac{b^{-1}(y)}{c^{-1}(y)} > \frac{c'(0)}{b'(0)}$$

(where $f'(x) = \frac{df}{dx}$).

The inverse of b and c are:

$$c^{-1}(y) = y^{1/h_c}$$

$$b^{-1}(y) = y^{1/h_b}$$

So

$$\begin{aligned} \frac{b^{-1}(y)}{c^{-1}(y)} &= y^{1/h_b - 1/h_c} \\ &= y^{\frac{h_c - h_b}{h_c h_b}} \end{aligned}$$

The derivatives of b and c with respect to σ_E are:

$$\begin{aligned} c'(\sigma_E) &= h_c \sigma_E^{h_c - 1} \\ b'(\sigma_E) &= h_b \sigma_E^{h_b - 1} \end{aligned}$$

and we can define $F(\sigma_E) \equiv \frac{c'(\sigma_E)}{b'(\sigma_E)} = \left(\frac{h_c}{h_b}\right) \sigma_E^{h_c - h_b}$. When $h_b < h_c$ we have $F(\sigma_E) \to 0$ for $\sigma_E \to 0$ and when $h_b > h_c$ that $F(\sigma_E) \to \infty$ for $\sigma_E \to 0$.

When $h_b < h_c$ we have thus have a function pair which falls in the continuous class since:

$$y^{\frac{h_c - h_b}{h_c h_b}} > 0$$

for all $0 < y$ and the optimal production curve $\sigma_E^{opt}(N)$ will thus be a continuous function. See figure 2A for an example of a function pair with $1 < h_b < h_c$ shown for different values of N. Note that the benefit function is here convex yet the optimal production curve will still be continuous since the cost curve is more convex than the benefit curve.

When $h_b > h_c$ there exists a value $y^* > 0$ such that

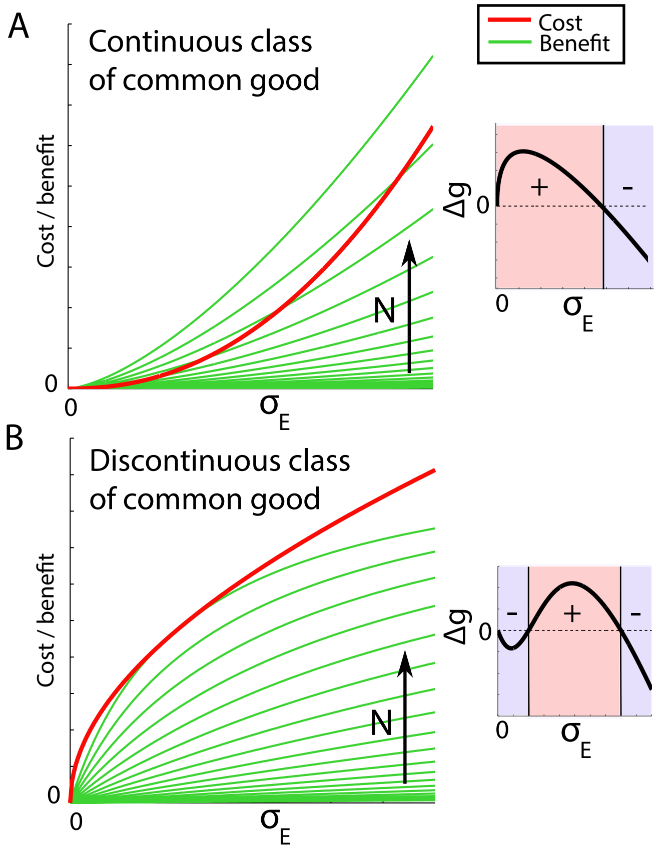

Figure 2: Red line: $c(\sigma_E) = \sigma_E^{h_c}$ and Green lines $b(N\sigma_E) = \sigma_E^{h_b}$ A: $(1 < h_b < h_c)$ An example of a cost benefit function pair which falls into the continuous class of common goods. Note that the benefit function is convex yet the optimal production curve will still be continuous since the cost curve is more convex than the benefit curve. B: $(0 < h_c < h_b < 1)$ An example of a cost benefit function pair which falls into the discontinuous class of common goods. Note that the benefit function is concave yet the optimal production curve will still be discontinuous since the cost curve is less concave than the benefit curve.

$$y^{*\frac{h_c-h_b}{h_c h_b}} < \infty$$

and the function pair thus falls into the discontinuous class which has a discontinuous optimal production curve $\sigma_E^{opt}(N)$. See figure 2B for an example of a function pair with $h_c < h_b < 1$ shown for different values of N. Note that the benefit function is here concave yet the optimal production curve will still be discontinuous since the cost curve is less concave than the benefit curve.

6 Relaxing the assumptions from the main text

6.1 Spatially structured systems

In the paper we deal with a well mixed system where where we can assume that concentration of common good E will be proportional to both production rate σ_E and population size N. However in a spatial system the common good produced by a cell in for example the center of the colony may not be able to diffuse fast enough before decaying to be available to all cells in the population. When the length scale of the colony size is much bigger than the average length which a common good molecule will diffuse,[1] the effective population size N_{eff} felt by a cell in the colony will be different than the actual population size, $N > N_{eff}$. How the effective population size N_{eff} depends on the actual population size N depends on dimensionality of the system n, common good degradation/depletion rate γ_E, and the diffusion rate D of the common good, (i.e. $N_{eff} = f(N, D, \gamma_E, n)$). In most cases N_{eff} will saturate at some finite value $\lim_{N\to\infty}(N_{eff}) = N_{max}$ as N goes to infinity. For a spatial system we will thus not have a common good concentration which is proportional to population size $E \propto \sigma_E N$ but rather $E \propto \sigma_E N_{eff}$, where $N_{eff} = f(N)$ is a saturating function of N. The optimal production rates will thus not fall to zero as $N \to \infty$ for a spatial system but rather converge towards the finite non-zero value of $\sigma_E^{opt}(N_{max})$.

In any spatial system we can write the benefit function as a function of the common good production rate σ_E and the effective population size $N_{eff} = f(N)$:

$$B_{N_{eff}}(\sigma_E) = b(N_{eff}\sigma_E) = b(f(N)\sigma_E)$$

We see that the results obtained for the well-mixed system are applicable to a spatial system, if we just replace N with a suitable choice of $N_{eff} = f(N, n, D, \gamma_E)$. Note that we can vary the effective population size $N_{eff} = f(N, n, D, \gamma_E)$, while keeping N constant by changing, for example, the diffusion constant of the common good, D. We thus see that there can be a diffusion constant D_{crit} for a fixed set of (N', n', γ'_E) corresponding to the critical effective population size above which common good production will not pay off and below which it will pay off, $N_{eff,crit} = f(N', n', \gamma'_E, D_{crit})$.

6.2 Not reaching steady-state

In the calculation in the main text, we used the assumption that cell growth and division occurs on a much slower timescale than public good production and degradation/depletion to replace E by $N\sigma_E/\gamma_E$. If this assumption is not true, then there would not be enough time for E to get to this steady-state level. For example, in a well-mixed system, suppose at time 0 the system had N cells and a public good concentration of $E(0)$, then if the next cell division happened at a time t:

$$E(t) = E(0)e^{-\gamma_E t} + \left(1 - e^{-\gamma_E t}\right)\frac{N\sigma_E}{\gamma_E}. \tag{4}$$

So, even though E has not reached steady-state, it is nevertheless some increasing function of σ_E and N. Clearly, even a modest separation of timescales, such that the cell growth rate is a few times smaller than γ_E, will result in $E(t)$ being almost proportional to $N\sigma_E$

[1] $L_E \sim n\sqrt{D/\gamma_E}$ where D is the diffusion rate, γ_E is the degradation/depletion rate of the common good and n the dimensionality of the system.

and the same conclusions as before. Only if the cell growth rate is comparable to γ_E will there be a significant deviation from our calculation above. But even in this case, $E(t)$ will nevertheless be some increasing function of σ_E and N, just not a linear function. In general, we expect that cell growth will always be a few times slower than the rate of degradation/depletion of the public good and, thus, there will be at most a minor modification of the shape of the optimal production curve.

7 The critical α_c where the quorum sensing ODE model displays bi-stability

The ODE model presented in the main text of a well mixed population producing QS signal and public good (eq. 4 and 5 in the main text) displays bi-stability in certain parts of the parameter space. We wish to determine the critical value of α_c above which the system will be bistable for a range of N values.

The rate of change of signal molecule is given by:

$$\frac{dS}{dt} = N\left(1 + \sigma_S^{max}\frac{S^\alpha}{S^\alpha + K_S^\alpha}\right) - S$$

at steady state $\frac{dS}{dt} = 0$, we have:

$$1 + \sigma_S^{max}\frac{S^\alpha}{S^\alpha + K_S^\alpha} = \frac{S}{N}. \tag{5}$$

We define

$$p(S) \equiv 1 + \sigma_S^{max}\frac{S^\alpha}{S^\alpha + K_S^\alpha}.$$

Fix points S^* are found when $p(S)$ intersect with S/N. The system will thus be bistable when it is possible for a straight line S/N (that passes through the origin and has a positive slope) to intersect with $p(S)$ for more than one value of S. Above a critical value of $\alpha = \alpha_c$ it becomes possible to get 3 intersections. (See fig. 3 where $p(S)$ is plotted for two different values of α (above and below the critical value), along with a range of straight lines, S/N).

We wish to determine the value α_c above which the system displays bi-stability.

We realize graphically that if a straight line l exists, which passes through a point $(S^*, p(S^*))$ and has a slope of $\frac{dp}{dS}\Big|_{S=S^*}$ can intersect the y-axis below zero, then eq. (5) can have three solutions. The equation for the line l is:

$$l: \quad p(S') = S'\frac{dp}{dS}\Big|_{S=S'} + q$$

where q is the intersection of l with the y-axis.

The first derivative of $p(S)$ is:

$$\frac{dp}{dS} = \alpha K_S^\alpha \sigma_S^{max}\frac{S^{\alpha-1}}{(S^\alpha + K_S^\alpha)^2}$$

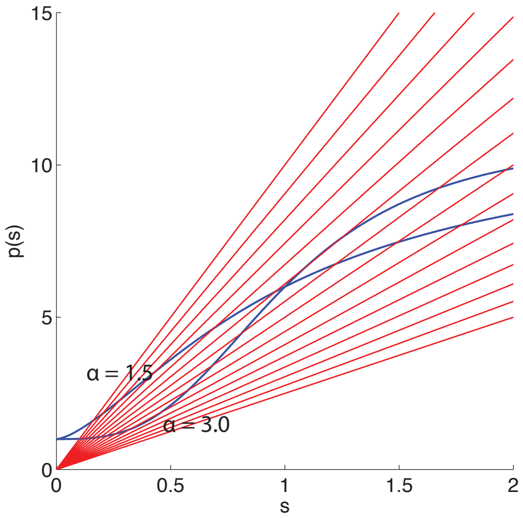

Figure 3: Blue lines shows $p(S)$ plotted for two different values of $\alpha = [1.5, 3.0]$, where the system can have only one or up to three fix points respectively. Red lines are S/N plotted fro a range of different N values (between 0.1 and 4.0). Fix points are values of S where $p(S)$ and S/N intersects. (Here $\sigma_S^{max} = 10$, and $K_s = 1$).

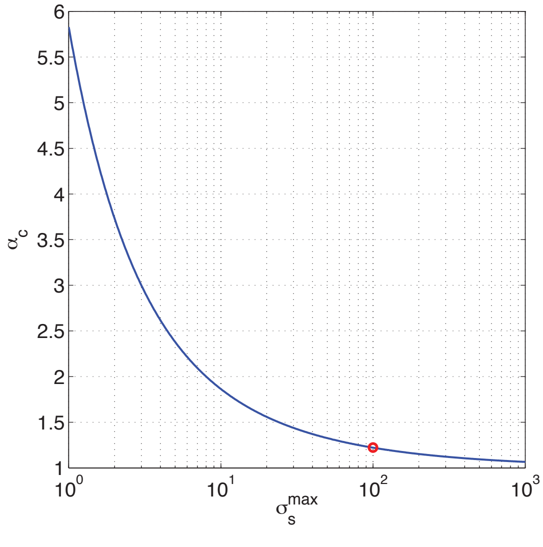

Figure 4: Blue line is $\alpha_c = \frac{2+\sigma_s^{max}+2\sqrt{1+\sigma_s^{max}}}{\sigma_s^{max}}$, (eq. 7) as a function of the ratio between basal and maximal signal production rates, σ_s^{max}. The red circles marks the value of σ_s^{max} used in the paper. The actual value of σ_s^{max} not well known for any bacterial species but it is reasonable to assume its lies in the range of $[5 - 1000]$ and possibly could be very different for different species.

For bi-stability (three fix points) we must thus have:

$$
\begin{aligned}
q &< 0 \Rightarrow \\
0 &> p(S') - S' \frac{dp}{dS}\Big|_{S=S'} \Rightarrow \\
0 &> 1 + \sigma_S^{max} \frac{S'^{\alpha}}{S'^{\alpha} + K_S^{\alpha}} - \alpha K_S^{\alpha} \sigma_S^{max} \frac{S'^{\alpha-1}}{(S'^{\alpha} + K_S^{\alpha})^2} S' \Rightarrow \\
0 &> 1 + \sigma_S^{max} \left[\frac{S'^{\alpha}}{S'^{\alpha} + K_S^{\alpha}} - \alpha K_S^{\alpha} \frac{S'^{\alpha}}{(S'^{\alpha} + K_S^{\alpha})^2} \right] \Rightarrow
\end{aligned}
\tag{6}
$$

We rename $S^{\alpha} \equiv x$ and rewrite eq. (6) to get a 2nd degree equation in x:

$$
\begin{aligned}
0 &> x^2 \left(1 + \sigma_S^{max}\right) + x K_S^{\alpha} \left(2 + \sigma_S^{max} (1 - \alpha)\right) + K_S^{2\alpha} \\
&\equiv x^2 a + x b + c
\end{aligned}
$$

there is exactly one solution $x_0 = S_0^{\alpha}$ to $x^2 a + xb + c = 0$ when the coefficients satisfy $d = b^2 - 4ac = 0$. This is when the line l will intersect the y-axis at zero at one value of $S^* \equiv S_0$ exactly (while for all other values of S^* the line l will intersect the y-axis above zero) and this defines the critical α_c above which the system starts to be bistable. This gives us the needed condition to find the critical α_c :

$$
\begin{aligned}
d &= 0 \Rightarrow \\
0 &= b^2 - 4ac \Rightarrow \\
0 &= \left(K_S^{\alpha} \left(2 + \sigma_S^{max} (1 - \alpha)\right)\right)^2 - 4 \left(1 + \sigma_S^{max}\right) K_S^{2\alpha} \Rightarrow \\
\alpha_{\pm} &= \frac{2 + \sigma_S^{max} \pm 2\sqrt{1 + \sigma_S^{max}}}{\sigma_S^{max}}
\end{aligned}
\tag{7}
$$

it is of course only the positive solution $\alpha_+ \equiv \alpha_c$ which is relevant. For α_- the solution x_0 to $x^2 a + xb + c = 0$ is negative ($x_0 < 0$) which we cannot have since $S \geq 0$.

Note that the critical point α_c is independent of K_S. This is expected since we can always choose the unit of S such that $K_S \equiv 1$.

8 Model for polymers degraded by an excreted endoprotease

An common example of an excreted public good are enzymes which act outside the cell to degrade long polymers into smaller pieces which subsequently can be transported over the cell membrane and metabolized. A well studied example is e.g. *Pseudomonas aeruginosa* which can excrete multiple endo-proteases [2] capable of degrading casein into casamino acids by breaking the polymers at sites of specific residues.

We wish to assess the shape of the benefit function for a well mixed environment with polymers that can be broken at random places by an excreted enzyme, (we assume that

[2] LasB and AprA, the two major secreted proteases by *Pseudomonas aeruginosa*.

the specific sites where the enzymes can break the polymers are distributed randomly. [3] We will assume that the fitness increase is proportional to the number of pieces of polymer present which are small enough for transport over the cell membrane. Thus what we need to determine is the steady state distribution of polymers of different lengths, and specifically the concentration of polymers of the "edible length", as a function of the concentration of excreted enzyme. We set this "edible length" (the maximal length that still allows transport over the cell membrane), to one, and we assume a constant external source of polymers of length n, and a constant degradation/depletion rate equal for polymers of all lengths.

Concentration of a polymer of length i is denoted N_i. Longest polymers (the ones supplied by an external source) in the system has length n. Concentration of enzyme (common good) is denoted E. The production rate of polymers of maximal length n, is p. Degradation/depletion rate of all polymers is δ.

Equations describing the change in concentration of polymers of all lengths, for a given level of enzyme, E, are thus as follows:

$$\begin{aligned}
\frac{dN_n}{dt} &= p - N_n(E+\delta) \\
&\vdots \\
\frac{dN_i}{dt} &= 2E\sum_{j=i}^{N-1}\frac{N_{j+1}}{j} - N_i(E+\delta) \\
&\vdots \\
\frac{dN_1}{dt} &= 2E\sum_{j=1}^{N-1}\frac{N_{j+1}}{j} - N_1\delta
\end{aligned}$$

where $i = 1, 2, \ldots, n$

The steady state concentrations, $N_n^*, \ldots, N_i^*, \ldots, N_1^*$ can be found by setting $\dot{N}_n = \cdots = \dot{N}_i = \cdots = \dot{N}_1 = 0$.

For $n = 2$, the steady state concentration of N_1 will be:

$$N_{1,n=2}^* = 2\frac{Ep}{\delta(\delta+E)} \tag{8}$$

and for $n > 2$, the steady state concentration of N_1 is given by:

$$N_{1,n>2}^* = \frac{2}{n-1}\frac{Ep}{\delta(\delta+E)}\left[1+\left(\frac{2}{n-2}+\sum_{k=2}^{n-2}\frac{1}{k-1}\prod_{j=k}^{n-2}\left(1+\frac{2}{j}\frac{E}{\delta+E}\right)\right)\frac{E}{\delta+E}\right] \tag{9}$$

Eq. (8) and (9) are plotted in figure 5. Note that it is only for a system with a maximal polymer length of $n = 2$ that the benefit function is *not* convex. (The second derivative of eq. (8) is negative while the second derivatives of eq. (9) are increasingly more positive for increasing n). This means that in general, benefits will accelerate with

[3]The proteases LasB and AprA secreted by e.g. *Pseudomonas aeruginosa*, are endoproteases, which means they cut the protein next to specific residues. Endoproteases are generally more common among QS-regulated secreted enzymes than exoproteases which cleaves polymers from the end.

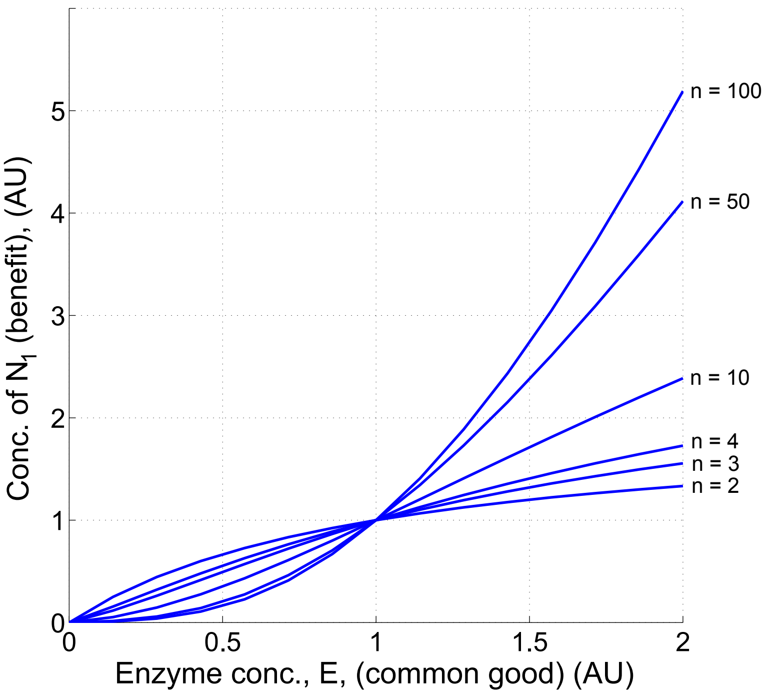

Figure 5: **Benefit function becomes increasingly more convex as length of longest polymer in system increases, when degradation is done by an exoprotease.** Steady state concentration of N_1 as a function of enzyme concentration E, for systems with max polymer length of $n = [2, 3, 4, 10, 50, 100]$.

increasing concentration of enzyme if the polymers provided by the external source has a length of more than two 'edible' units (i.e. units small enough to be transported over the cell membrane).

Expression eq. (9) comes from combining the fact that:

$$N_1^* = 2\frac{E}{\delta}\left[\sum_{k=2}^{n}\frac{1}{k-1}N_k^*\right], \text{ for } n > 2$$

and

$$\begin{aligned} N_i^* &= N_{i+1}^*\left(1+\frac{2}{i}\frac{E}{\delta+E}\right), \text{ for } 1 < i < n-1 \Rightarrow \\ N_k^* &= N_{n-1}^*\prod_{j=k}^{n-2}\left(1+\frac{2}{j}\frac{E}{\delta+E}\right), \text{ for } k > 1 \end{aligned}$$

and

$$\begin{aligned} N_n^* &= \frac{p}{\delta+E} \\ N_{n-1}^* &= \frac{2}{n-1}\frac{E}{\delta+E}N_n^*, \text{ for } n > 2 \end{aligned}$$

which follows from setting $\dot{N}_n = \cdots = \dot{N}_i = \cdots = \dot{N}_1 = 0$, and solving for $N_n^*, \ldots, N_i^*, \ldots, N_1^*$.

9 Common microbial public goods and a discussion on the possible shape of their benefit functions

9.1 Virulence factors

It has been proposed that when virulent bacteria delay production of a virulence factor, via QS regulation, it is because a low concentration of the virulence factor would alert the host immune system, while at the same time not do much harm to the host. Instead, delaying production of the virulence factor until a sufficiently high bacterial density had been reached could lead to more positive outcomes (from the bacterial point of view): either 1) death of the host organism on a faster time scale than the immune system could detect and launch a counterattack, or 2) enough time for the bacteria to produce means of protection (e.g. biofilm) from the immune system before producing virulence factors. This idea is sometimes referred to as the "sneak attack" hypothesis (e.g. in [2]) for obvious reasons. If this theory it is right, it is a good example of a situation where the benefit function of a common good (the virulence factor) would be highly convex. Note that in this scenario, as small amounts of virulence factor would provoke an attack from an otherwise indifferent immune system, the benefit could actually be negative for small concentrations of virulence factor.

9.2 Antimicrobials

Another typical common good is an antibiotic produced to kill or harm other species of bacteria living in the same habitat as the common good producer. It has been shown experimentally that the 'killing curve' - the rate of death of the bacteria which is sensitive to the antibiotics vs. the concentration of the antibiotics - is often sigmoidal with a hill factor of 2-4, ([1, 3]). This thus constitutes another example of a situation where the benefit function is initially convex. The source of the convexity of the killing-curve is not well-established. For toxins which damage parts of the target cell which are monitored by specific repair mechanisms, the convexity could be related to the time scale over which the cell-repair mechanisms act. Small doses of toxin may allow time for the repair mechanisms to 'keep up' and continuously repair the damage done by the toxin between each new damage event, while at higher concentrations of toxin the damage would start to accumulate and become fatal. Another factor, acting on an evolutionary time scale, is that small sub-lethal doses of antibiotics would allow an enemy bacterium to slowly adapt and become resistant to the toxin over time. On a more hypothetical note we can mention that an excreted common good molecule, which acted in a cooperative manner outside the cell (as is the case for many proteins acting inside the cell), would also result in a convex benefit function, although we do not currently know of any common goods where this has been demonstrated.

References

[1] Johan W Mouton, N. P., and Vinks, A. A. Concentration-effect relationship of ceftazidime explains why the time above the mic is 40 percent for a static effect in vivo. *Antimicrobial agents and chemotherapy 51*, 9 (2007), 3449–51.

[2] Winzer, K., and Williams, P. Quorum sensing and the regulation of virulence gene expression in pathogenic bacteria. *International journal of medical microbiology IJMM 291*, 2 (2001), 131–143.

[3] Yanjun Li, M Hong Nguyen, S. C. S. S. L. Z. H. D., and Clancy, C. J. A pharmacokinetic/pharmacodynamic mathematical model accurately describes the activity of voriconazole against candida spp. in vitro. *International journal of antimicrobial agents 31*, 4.
